# The complete mitochondrial genome and phylogenetic analysis of *Matuta planipes* (Decapoda: Brachyura: Matutidae)

**DOI:** 10.1080/23802359.2018.1437802

**Published:** 2018-02-07

**Authors:** Fan Lin, Huaqiang Tan, Hanafiah Fazhan, Zhuofang Xie, Mengyun Guan, Xi Shi, Hongyu Ma

**Affiliations:** Guangdong Provincial Key Laboratory of Marine Biotechnology, Shantou University, Shantou, China

**Keywords:** Matuta planipes, mitochondrial genome, phylogeny

## Abstract

The complete mitochondrial genome of *Matuta planipes* was obtained using long and conventional PCR method. The circular genome was 15,760 bp in length, consisting of 13 protein-coding genes, 22 transfer RNA genes, 2 ribosomal RNA genes and a control region. Of the 37 genes, 23 were encoded by the heavy strand, while the others were encoded by the light strand. The genome composition with A + T bias (70.82%) and gene arrangement were largely identical to those observed in most arthropods, such as the mud crab (*Scylla paramamosain*). The phylogenetic analysis suggested that *M. planipes* was closest to *Ashtoret lunaris.* The newly described mitochondrial genome may provide valuable data for phylogenetic analysis for Matutidae.

The flower moon crab*, Matuta planipes* mainly distributed in the Indo-Pacific region, is a species of the family Matutidae (de Grave et al. 2009). Different from other swimming crabs, the species in this family have five pairs of flattened legs (adapted for swimming and digging). Although placed in Calappoidea, the affinities of the Matutidae with the Calappidae are still not clear and are probably not closely related with each other (Ng et al. 2008). It has been suggested that Matutidae was more closely related with Leucosiidae than Calappidae, and should be placed in Leucosioidea (Bellwood 1996; Silambarasan et al. 2014). The complete mitochondrial genome sequences can facilitate the validation of taxonomic classification and population genetic structure (Liu and Cui 2010). However, rare mitochondrial genomes are currently available in the GenBank database, except for one Matutidae species (*Ashtoret lunaris*) and one Leucosiidae species (*Pyrhila pisum*). Therefore, more mitochondrial genome sequences of related species are needed. Here, the first complete mitochondrial genome DNA sequence of the *M. planipes* was reported and used for phylogenetic analysis of the species.

Specimens of *M. planipes* were collected from a fishing port in Yinggehai Town (18.5049°N, 108.6883°E), Hainan, China and kept in the Marine Biology Institute, Shantou University, Shantou, China. Total genomic DNA was isolated from the muscle tissue. Long and conventional PCR were employed to obtain the complete mitochondrial genome sequence.

The complete mitogenome sequence of *M. planipes* was 15,760 bp in length (Genbank accession number: MG756601). The gene composition and arrangement were largely identical to those observed in most arthropods, such as the mud crab (*Scylla paramamosain*) (Ma et al. 2013) and *Charybdis feriata* (Ma et al. 2015). The overall nucleotide composition of this genome was A + T biased (70.82%). The genome contained 13 protein-coding genes, 22 transfer RNA genes, 2 ribosomal RNA genes and 1 putative control region. Of the 37 genes, 23 were encoded by the heavy strand, and the others were encoded by the light strand. Eight protein-coding genes were initiated by ATG, and three genes (ND1, ND2 and ND6) were started by GTG. ATP6 and ND3 were started by ATT and ATA, respectively. Two kinds of termination codon (TAA and TAG) were identified in ten protein-coding genes, while three incomplete termination codons (T–) were found in the other three genes (COIII, ND5 and Cytb).

The phylogenetic tree was constructed based on 12 concatenated protein-coding genes (except ND6) from 36 crab species from Genbank database, by maximum likelihood (ML) method. *Hapiosquilla harpax* was used as an outgroup for tree rooting (Figure 1). It was demonstrated that *M. planipes* was clustered with *A. lunaris*, which belongs to Matutidae as well. Meanwhile, the two Matutidae species had far relationship with the Leucosiidae species *Pyrhila pisum*, indicating that these two may not be placed in Leucosioidea. However, more related complete mitochondrial genome is needed to verify the inference.

**Figure 1.**
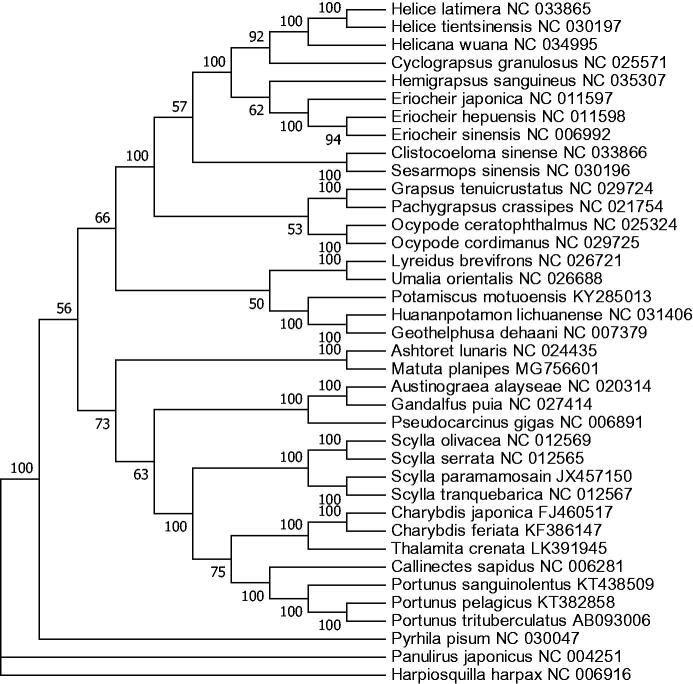
Phylogenetic tree of *M. planipes* and related species based on maximum likelihood (ML) method. *Hapiosquilla harpax* was used as an outgroup.
